# NLRP3 Inflammasome in Vascular Dementia: Regulatory Mechanisms, Functions, and Therapeutic Implications: A Comprehensive Review

**DOI:** 10.1111/cns.70403

**Published:** 2025-05-06

**Authors:** Yujia Lu, Lin Cheng, Yinyi Xiong, Chunyan Huang, Ziying Liu, Chunxiao Shen, Huaying Wang, Yuemin Qiu, Seung Bum Yang, Moxin Wu, Xiaorong Zhang

**Affiliations:** ^1^ Department of Pathology Clinical Medical School of Jiujiang University Jiujiang Jiangxi China; ^2^ Jiujiang Clinical Precision Medicine Research Center Jiujiang Jiangxi China; ^3^ Department of Neurology Clinical Medical School of Jiujiang University Jiujiang Jiangxi China; ^4^ Department of Rehabilitation Clinical Medical School of Jiujiang University Jiujiang Jiangxi China; ^5^ Department of Paramedicine Wonkwang Health Science University Iksan Republic of Korea

**Keywords:** inflammasome, NLRP3, stroke, vascular dementia

## Abstract

**Background:**

Vascular dementia, the second most common type of dementia globally after Alzheimer's disease, is associated with neuroinflammation. Activation of the NLRP3 inflammasome, an important pattern recognition receptor in human innate immunity, plays a key role in the pathogenesis of vascular dementia.

**Results:**

The NLRP3 inflammasome pathway destroys neuronal cells primarily through the production of IL‐18 and IL‐1β. Moreover, it exacerbates vascular dementia by producing IL‐18, IL‐1β, and the N‐terminal fragment of GSDMD, which also contributes to neuronal cell death. Thus, blocking the NLRP3 inflammasome pathway presents a new therapeutic strategy for treating vascular dementia, thereby delaying or curing the disease more effectively and mitigating adverse effects.

**Conclusions:**

This review explores the role and mechanisms of the NLRP3 inflammasome in vascular dementia, summarizing current research and therapeutic strategies. Investigating the activation of the NLRP3 inflammasome can reveal the pathogenesis of vascular dementia from a new perspective and propose innovative preventive and treatment strategies.

## Graphical abstract

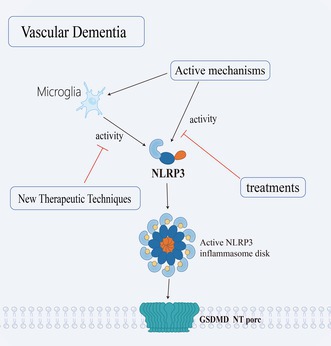

**Abbreviations**
AD: Alzheimer's disease
CCA: common carotid artery
CCH: chronic cerebral hypoperfusion
DAMP: damage‐associated molecular patterns
LRR: leucine‐rich repeat
MCAO: middle cerebral artery occlusion
MSN: magnesium silicide nanosheets
OMV: outer membrane vesicles
PAMP: pathogen‐associated molecular patterns
PD: Parkinson's disease
ROS: reactive oxygen species
TLR: toll‐like receptors
TNF: tumor necrosis factor
TNFR: tumor necrosis factor receptor

## 1 Introduction

Vascular dementia (VaD) is the second most prevalent type of dementia globally after Alzheimer's disease (AD), causing memory and cognitive deficits and negative emotional responses. VaD is more common in people over 65 years old and is associated with gender, blood pressure, blood glucose, and body weight [1, 2, 3]. VaD prevalence accounts for about 15%–20% of dementia cases in North America and Europe, and approximately 30% in Asia and developing countries [1, 4]. VaD prevalence is gradually increasing with the increasing age of the population, drawing considerable scholarly attention to its pathogenesis and treatment.

Despite ongoing research, the mechanisms underlying its cure remain incompletely understood. Recent advancements have identified the inflammasome, a protein complex crucial for cellular communication in VaD [5, 6]. The NLRP3 and AIM2 inflammasomes may play key roles in VaD, prompting further exploration of their mechanisms. Furthermore, neuroinflammation is associated with decreased cognitive function and functional connectivity in patients with dementia [7, 8], presenting with higher levels of inflammatory markers in brain and peripheral tissues [9]. Given the role of the NLRP3 inflammasome as a signaling pathway in neuroinflammation, its study is vital for understanding and potentially mitigating VaD.

This review summarizes the potential mechanisms of the NLRP3 inflammasome and the current methods and drugs in VaD. It focuses on the activation mechanisms of the NLRP3 inflammasome in VaD and integrates the newest technology (such as nanoparticles) to further analyze and summarize treatment approaches. We aim to pave the way for future research on the NLRP3 inflammasome in VaD.

## 2 Overview of VaD

Chronic cerebral hypoperfusion (CCH) due to cerebrovascular disease (including ischemic and hemorrhagic strokes) is now considered the primary cause of VaD [10, 11, 12, 13]. These diseases lead to reduced cerebral blood flow and impaired blood–brain barrier, which can easily lead to damage to brain structures including the hippocampus [14], lipid peroxidation, and neuronal death, which in turn reduces blood oxygenation, resulting in immune disorders, malnutrition, and metabolic weakness, ultimately triggering and exacerbating VaD [8, 15].

The mechanisms underlying VaD encompass neuroinflammation, oxidative stress, blood–brain barrier dysfunction, neurovascular nutrient decoupling, nerve cell demyelination, and mitochondrial impairment (morphological and functional) [16]. These processes lead to pathological alterations such as apoptosis [6, 8, 16]; the NLRP3 inflammasome can exacerbate neuroinflammation and aggravate brain disorders [17]. Moreover, mice lacking NLRP3 exhibit reduced infarct volume and decreased neuronal death after cerebral ischemia, which indicates that the NLRP3 inflammasome plays an important role in VaD [18, 19]. Thus, what is the NLRP3 inflammasome?

## 3 Overview of the NLRP3 Inflammasome

The inflammasome contributes to the innate immune response, consisting of cytoplasmic protein complexes that can mediate the activation of potent inflammatory mediators. When cells are infected by bacteria and viruses, they promote the expression, maturation, and release of various pro‐inflammatory cytokines, thereby triggering a series of immune responses [20, 21, 22]. The NLRP3 inflammasome belongs to the NLR family, which is involved in many bodily reactions and is important for maintaining human health homeostasis [23].

### 3.1 Structure of the NLRP3 Inflammasome

The NLRP3 inflammasome is produced by the bone marrow macrophages [24]. Upon stimulation, it assembles and activates caspase‐1, which induces a series of reactions promoting cell death [22, 25, 26, 27, 28, 29]. The NLRP3 inflammasome is a key driver of vascular diseases, including atherosclerosis [30, 31]. This inflammasome comprises NLRP3 (innate immunity receptor protein), ASC (articulation), and Caspase‐1 (inflammatory protease cysteinyl asparagine‐1). These proteins are folded into a typical cyclic conformation, which can be divided into three structural domains: the n‐terminal pyrin (PYD), the central NACHT, and the c‐terminal leucine‐rich repeat (LRR) [25, 26, 32, 33]. One of the PYD structural domains is hidden within the membrane‐bound NLRP3 oligomeric bicyclic cage (the membrane is an oligomeric scaffold) to avoid premature activation of NLRP3 [34]. This structure features five connecting loops and six helices (α1–α6), folded in a typical antiparallel helix bundle and tightly wrapped to form a hydrophobic core [35]. The unique disulfide bond formed by conserved residues in NLRP3, C8 (α1 helix), and C108 (the loop connecting PYD and NACHT) may contribute to reactive oxygen species (ROS) signaling and NLRP3 inflammasome activation [20], ultimately causing cellular pyroptosis [25].

### 3.2 Activation of the NLRP3 Inflammasome

The binding of NLRP3 to its receptor on the cell membrane activates the NLRP3 inflammasome, releasing inflammatory factors such as interleukins and inducing inflammatory responses in the organism [36].

In the resting state, NLRP3 presents a specific conformation in which the LRR domain is inactive and cannot effectively recognize or bind to PAMPs or DAMPs [37]. Upon stimulation by microbial and non‐microbial factors such as bacterial toxins, particulates, and lipopolysaccharides (LPS) [38], NLRP3 may be involved in potassium ion efflux [38, 39, 40], lysosomal damage and membrane instability due to entosis of sterile particles, ATP binding to the purinergic P2X7 receptor (P2X7R) [40, 41, 42, 43], ionic abnormalities, and ROS, leading to its activation [24, 44, 45, 46]. As intracellular pH decreases, NLRP3 conformation changes, allowing the LRR domain to rearrange and recognize these activation signals.

**FIGURE 1:**
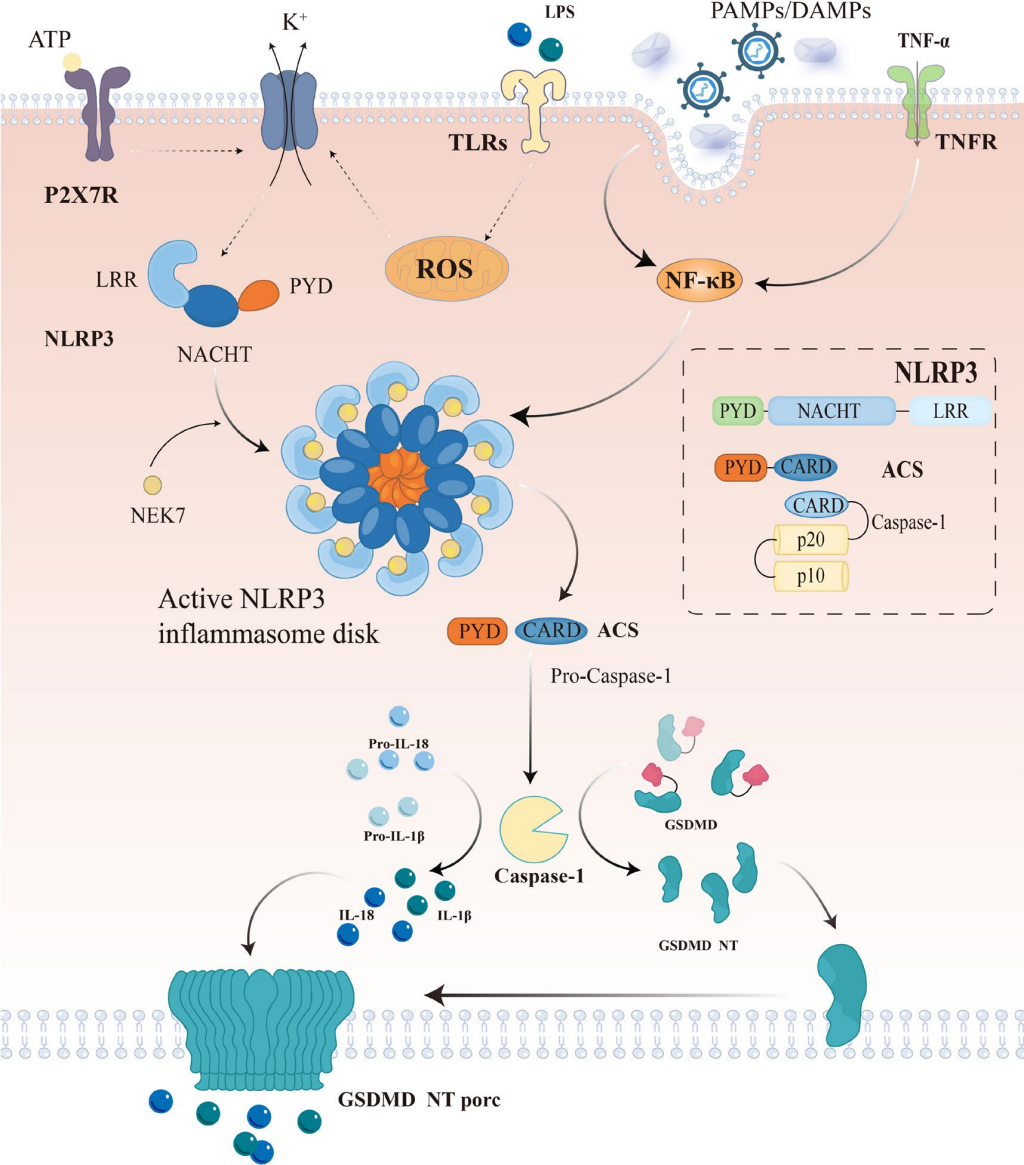
Activation mechanism of the NLRP3 inflammasome. When the external environment changes or is stimulated by other factors, NLRP3 gets activated. Initially, the conformation of NLRP3 changes, leading to LRR domain rearrangement and binding to NEK7. Thus, PYD domains interact with each other, recruiting caspases. This process ultimately generates the N‐terminal fragment of the GSDMD, which destroys cells by forming membrane pores and producing inflammatory factors such as IL‐18 and IL‐1β, promoting cellular pyroptosis.

Once bound, NLRP3's conformation is further stabilized, forming a platform capable of interacting with other proteins [47, 48]. The NLRP3 inflammasome oligomerizes through the NACHT domain to form the NLRP3 protein complex [49] and recruits the ASC protein through the PYD–PYD domain interaction to form the NLRP3–ASC complex [50]. Subsequently, caspase‐1 is recruited into the NLRP3–ASC complex, whereas its CARD domain interacts with the CARD domain of ASC, allowing caspase‐1 to approach and immobilize the active centers of NLRP3 and ASC, thus forming a complete NLRP3 inflammasome [35]. In this complex, pro‐caspase‐1 is cleaved into active caspase‐1, which then cleaves pro‐IL‐1β and pro‐IL‐18 into their mature forms. These inflammatory factors are then released outside the cell, triggering an inflammatory response [51]. Additionally, the activation of NLRP3 leads to the cleavage of gastrin D (GSDMD), production of active N‐terminal fragments of GSDMD, and cell membrane disruption with pore formation, which causes cell apoptosis, mitochondrial damage, and mitochondrial autophagy inactivation [52, 53, 54] (Figure 1).

## 4 Role of the NLRP3 Inflammasome in VaD

The NLRP3 inflammasome contributes to the mechanisms of many diseases, particularly metabolic disorders [55] and central nervous system diseases, such as VaD, stroke [56], AD [57, 58], type II diabetes mellitus [55], atherosclerosis [59], obesity [60], and gout [55]—due to vascular causes—as well as certain autoimmune diseases [61]. In AD, many studies and models (e.g., APP/PS1 mice or Tg2576 mice) have demonstrated the activation of the NLRP3 inflammasome [62, 63, 64]. Its pathology involves the accumulation of Aβ plaques and the development of intracellular neuroprogenitor fiber tangles, which are closely related to the activation of the NLRP3 inflammasome [65, 66]. Aβ produced during microglia activation can activate the NLRP3 inflammasome [67], and the activation of the NLRP3 inflammasome releases inflammatory factors (e.g., IL‐1β and IL‐18) outside the microglia. This results in the overexpression of IL‐1β in the brains of patients with AD and overphosphorylation of tau proteins, which together exacerbate neuronal damage and neurofibrillary tangle formation [68, 69, 70, 71, 72, 73, 74].

The NLRP3 inflammasome plays a key role in the pathogenesis of VaD, with immune cells (such as microglia [5] and astrocytes), cytokines, and chemokines being key players in neuroinflammation and the onset and development of VaD [75, 76]. When the damage signal is recognized by immune cells in the central nervous system, the inflammasome is activated, inducing neuroinflammation, with a series of immune cascade reactions, causing cell damage [66, 77, 78]. In stroke and early stages of VaD, the NLRP3 inflammasome action eventually leads to pyroptosis [79], promoting stroke [80, 81]. Our review focuses on how the overexpression of NLRP3 inflammasomes leads to vascular damage, contributing to VaD [82].

### 4.1 Neuroinflammation Exacerbated by the NLRP3 Inflammasome in VaD

The activation of the NLRP3 inflammasome initiates and exacerbates neuroinflammation [81]. Following cerebrovascular diseases (including ischemic and hemorrhagic strokes), cerebrovascular vessels are damaged, impairing blood circulation and leading to CCH, local cerebral tissue ischemia, and hypoxia [17, 83]. This condition is further stimulated by ROS, an imbalance of ion homeostasis (K^+^ efflux and Ca^2+^ influx) [84], mitochondrial dysfunction, and lysosomal rupture [38, 84, 85]. The NLRP3 inflammasome initially detects activation signals through specific receptors, leading to its activation and assembly [35], then the production and development of neuroinflammation. This process involves oligomerization, ASC recruitment [86], NLRP3–ASC complex formation, and the maturation and release of IL‐1β and IL‐18 [29], along with GSDMD lysis [57, 87].

The inflammatory factors (including IL‐1β) released upon activation of the NLRP3 inflammasome pathway can maintain local inflammation in the brain. GSDMD‐n, the lysate product of GSDMD, can destroy cell membranes to form membrane pores, promoting the damage of neurons and other cells, and leading to cognitive impairment and VaD progression [88]. Interleukin‐1 (IL‐1) impairs cerebral endothelial cells and neurons, reducing blood–brain barrier (BBB) permeability, compromising neural function, and increasing cerebrovascular activation [88]. This occurs primarily through IL‐1β/IL‐1R1/TRAF6 signaling, which promotes microglial activation and subsequent damage to adjacent neurons [89]. In intracerebral hemorrhage (ICH) mouse models, IL‐18 has been shown to interact with the sodium‐potassium‐chloride co‐transporter 1 (NKCC1) via complex formation, amplifying microglia‐mediated neuronal injury [90]. These reactions can attract other immune cells and inflammatory metabolites to the injured tissue, resulting in re‐occlusion of blood vessels, further aggravating brain tissue ischemia and nerve damage, and perpetuating a vicious cycle that worsens dementia and other symptoms [30].

### 4.2 Cells Contributing to the NLRP3 Inflammasome Activity in VaD

#### 4.2.1 Microglia

In the early stages of neuroinflammation, microglia, which detect and respond to abnormalities in the brain, are rapidly activated [80]. These cells are widely distributed in the brain and act as the main innate immune cells and the first responders to pathological damage [68, 81, 91, 92]. Microglia can polarize into M1 and M2 under different stimuli, increasing the production of pro‐inflammatory cytokines, ROS, iNOS, and COX, thereby triggering the formation of inflammatory bodies. This pro‐inflammatory function plays an important part in the mechanism of many neurodegenerative diseases, such as VaD [93, 94], AD [95, 96, 97], and Parkinson's disease [98, 99, 100].

**FIGURE 2:**
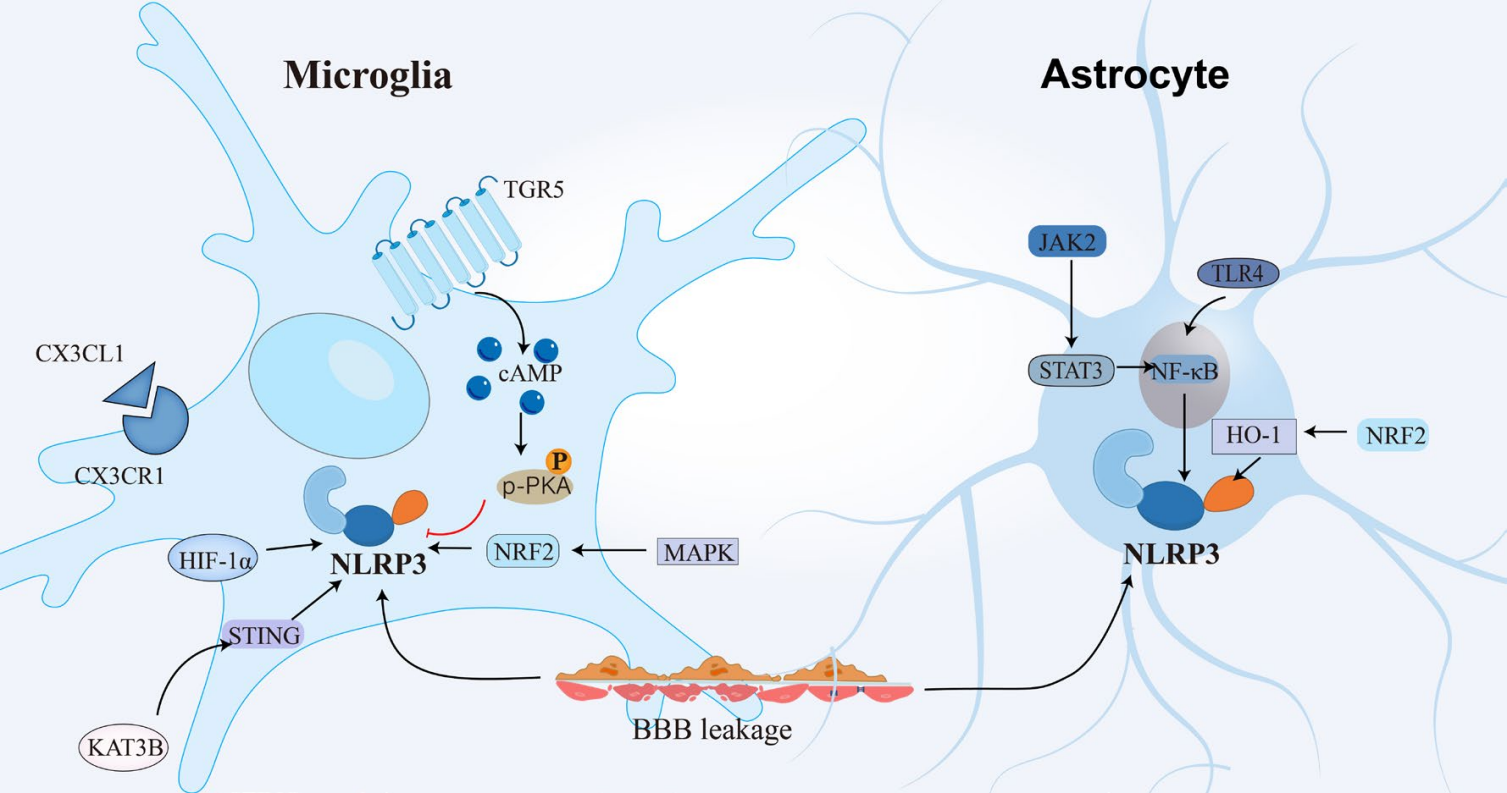
The NLRP3 inflammasome activation pathway. In microglia, the TGR5/cAMP/PKA, KAT3B‐STING, and CX3CL1 (CX3CL1 and its receptor CX3CR1) signaling pathways and HIF‐1α influence the NLRP3 inflammasome activation pathway. In astrocytes, the TLR4/NFκB/NLRP3 and JAK2/STAT3 signaling pathways are involved.

The NLRP3 inflammasome is involved in microglial activation through the miR‐143/PUMA axis; however, the specific mechanism remains unclear [101]. The overactivated microglia release pro‐inflammatory factors such as NO, interferon‐γ, tumor necrosis factor‐α, and IL‐1β through the NLRP3 inflammasome, aggravating ischemic injury and sustaining inflammation in brain lesions [30, 98] (Figure 2). Additionally, it is interesting that microglia can inhibit neuroinflammation through autophagy [98] or PGC‐1α [102], suggesting that the NLRP3 inflammasome is involved in microglial activation with a crucial role in VaD.

#### 4.2.2 Astrocytes

When microglia are activated during chronic CNS inflammation, the astrocytes become activated and proliferate, promoting the recovery of injured nerves. However, with disease development, astrocytes may over‐proliferate and release many excitatory neurotransmitters, such as glutamate, causing abnormal activity of synaptic potentials [103, 104]. Additionally, astrocytes can widely mediate the secretion of neuroinhibitory factors by inflammatory mediators, forming glial scars, which hinder brain tissue repair and further damage the nervous system [67].

In microglia, the NF‐κB pathway activates the NLRP3 inflammasome, thereby activating caspase‐1 to induce the secretion of neurotoxic astrocyte inducers. This results in the production of neurotoxic astrocytes, thereby aggravating neuroinflammation and causing neuronal loss [105]. Expanding on the role of the NLRP3 inflammasome in VaD, Yiwei Liu et al. [106] presented another therapeutic perspective. Their study reports that osthole enhances cognitive functions in VaD rat models while reducing Aβ deposition, an effect closely associated with the inhibition of NLRP3 inflammasome‐induced microglial activation.

The inflammatory factors released by microglia and astrocytes may damage neurons, leading to cognitive impairment, or attract other immune cells and inflammatory metabolites to the injured tissue, exacerbating nerve damage and creating a vicious cycle [107]. This evidence suggests that microglia and astrocytes play a critical role in NLRP3‐mediated neuroinflammation of VaD and highlights NLRP3 as a key therapeutic target for VaD.

### 4.3 Possible Regulatory Pathways of the NLRP3 Inflammasome in VaD

Recent research has highlighted the role of the NLRP3 inflammasome in the pathophysiology of cognitive impairment‐related diseases, including VaD, highlighting potential interventions that could ameliorate these conditions [108, 109]. However, the signaling pathway remains unclear. Current studies are sporadic, without an established unified view. However, these studies are crucial for the development of targeted drugs. What are the pathways involved in the NLRP3 inflammasome?

In the middle cerebral artery occlusion (MCAO) mouse model and the in vitro oxygen–glucose deprivation/reoxygenation (OGD/R) model, the JAK2/STAT3, KAT3B‐STING, AMPK/Nrf2/NLRP3, and CX3CL1 (CX3CL1 and its receptor CX3CR1) signaling pathways affected the activation and initiation of the NLRP3 inflammasome. Ruxolitinib (Rux) can diminish the phosphorylation of STAT3 through the JAK2/STAT3 pathway, suppress the NLRP3 promoter, and alleviate the inflammatory response [110]. Additionally, methyltransferase‐like 14 (METTL14) can restrain NLRP3 inflammasome activation via the KAT3B‐STING signal transduction axis [111]. Bakuchiol (BAK) can restrain NLRP3 inflammasome activation via the AMPK/Nrf2/NLRP3 signal transduction axis [112]. The CX3CL1 signaling pathway (CX3CL1 and its receptor CX3CR1) can also prevent microglial pyroptosis induced by the NLRP3 inflammasome [113]; preclinical experiments have demonstrated that this pathway impacts functional restoration after stroke [114].

Moreover, the TLR4/NFκB/NLRP3 signaling pathway, a key route for NLRP inflammasome therapy, has shown therapeutic effects in animal studies on liver conditions and atherosclerosis. This pathway might be relevant in patients with VaD, where repeated transcranial magnetic stimulation (rTMS) has been demonstrated to modulate microglial polarization, thereby alleviating dyskinesia and neuronal pyroptosis caused by brain I/R injury.

Moreover, the TLR4/NFκB/NLRP3 signaling pathway, a key route for NLRP inflammasome therapy, has shown therapeutic effects in animal studies on liver conditions [115] and atherosclerosis [116]. This pathway might be relevant in patients with VaD, where repeated transcranial magnetic stimulation (rTMS) has been demonstrated to modulate microglial polarization, thereby alleviating dyskinesia and neuronal pyroptosis caused by brain I/R injury [117]. Within this pathway, Toll‐like receptor 4 (TLR4) recognizes proinflammatory stimuli [118, 119]. Upon activation, the TIR domain of TLR4 interacts with the TIR domain of myeloid differentiation primary response 88 (MyD88), inducing conformational changes in MyD88 that expose its death domain, forming a MyD88–IRAK4–IRAK1 complex that activates downstream TNF receptor‐associated factor 6 (TRAF6), which ultimately triggers nuclear translocation of NF‐κB [120, 121]. NF‐κB activation further facilitates NLRP3 inflammasome assembly, initiating canonical inflammatory cascades. Recent studies in MCAO rat models have validated this mechanism, demonstrating that 4‐methylumbelliferone (4‐MU) attenuates injury through pathway inhibition [122]. These findings elucidate the regulatory network of NLRP3 and establish a theoretical foundation for targeted therapeutic strategies.

In brief, the NLRP3 inflammasome plays a central role in VaD by exacerbating neuroinflammation and neuronal apoptosis through various mechanisms. The main mechanism of NLRP3 in VaD may be related to JAK2/STAT3/NLRP3, AMPK/Nrf2/NLRP3, and TLR4/NFκB/NLRP3 signaling pathways. We should further focus on the specific mechanisms and regulatory pathways of its onset and development, as well as explore its therapeutic potential and clinical applications in the future.

### 4.4 Interactions Between the NLRP3 Inflammasome and Other Inflammasome

Current research has found that the NLRP3 inflammasome exhibits synergistic effects with many other inflammasomes, including NLRP3, AIM2, NLRC4, and Pyrin inflammasomes [47]. Although inflammasome sensors are specific, the disease progression may generate multiple PAMPs/DAMPs that simultaneously activate various inflammasomes to work cooperatively. When NLRP3, AIM2, NLRC4, and Pyrin are activated concurrently, they can react with ASC, caspase‐1, caspase‐8, and RIPK3 to form a large multiprotein complex, inducing various forms of programmed cell death. Additionally, when these large multiprotein complexes are released into the extracellular space, they can function as multiple inflammasomes [26, 47, 123].

However, experiments focusing on the interactions between inflammasomes in VaD remain limited. Nevertheless, this undeniably represents a fascinating research entry point. Such investigations will not only reveal the unique status of NLRP3 within inflammatory networks but also clarify how its synergistic mechanisms promote VaD pathological processes. We look forward to future experiments and clinical evidence validating these inflammasome interactions in VaD.

### 4.5 Role of the NLRP3 Inflammasome in VaD‐Related Diseases

We have discovered that the NLRP3 inflammasome in other related diseases, such as hypertension and diabetes, also influences VaD. Hypertension increases the risk of VaD by elevating blood pressure, leading to hypoxia, and promoting neuroinflammation. Inflammation in hypertension involves numerous immune cells; immune activation can be triggered by neoantigens, NLRP3 inflammasomes, cytokines (including IL‐6, IL‐7, IL‐15, IL‐18, and IL‐21), and a high salt environment. These inflammatory mediators also facilitate the onset of VaD, which can lead to microvascular thinning and dysfunction, as well as neurovascular uncoupling and damage to the brain blood supply [75, 124]. While patients with type 2 diabetes (T2D) also experience cognitive decline and a higher risk of VaD due to neuroinflammation, vascular degeneration, and glial activation, with the NLRP3 inflammasome playing an important role in neuroinflammation [125]. These studies suggest that the NLRP3 inflammasome could be a crucial target for VaD treatment. Additionally, new technologies, such as nanomaterials [126] and hydrogen [127], have shown promise in improving VaD symptoms in animal studies, supporting the feasibility of this target.

## 5 Strategies for Treating VaD by Targeting the NLRP3 Inflammasome

The NLRP3 inflammasome pathway can lead to neuroinflammation, a mechanism that contributes to VaD. Blocking this pathway may help treat VaD [128, 129, 130, 131]. Two stages can be blocked: First, by preventing the initiation and activation of the NLRP3 inflammasome, and second, by influencing the secreted factors or cell interactions within this pathway [132].

### 5.1 Blocking the Activation of the NLRP3 Inflammasome

For the NLRP3 inflammasome to function, it must first be stimulated and activated by specific substances before undergoing complex structural changes [29]. Treating inflammation caused by the NLRP3 inflammasome involves blocking the activation and assembly of the NLRP3 inflammasome. Currently, many therapeutic mechanisms, drugs, and approaches have been discovered [132, 133].

**FIGURE 3:**
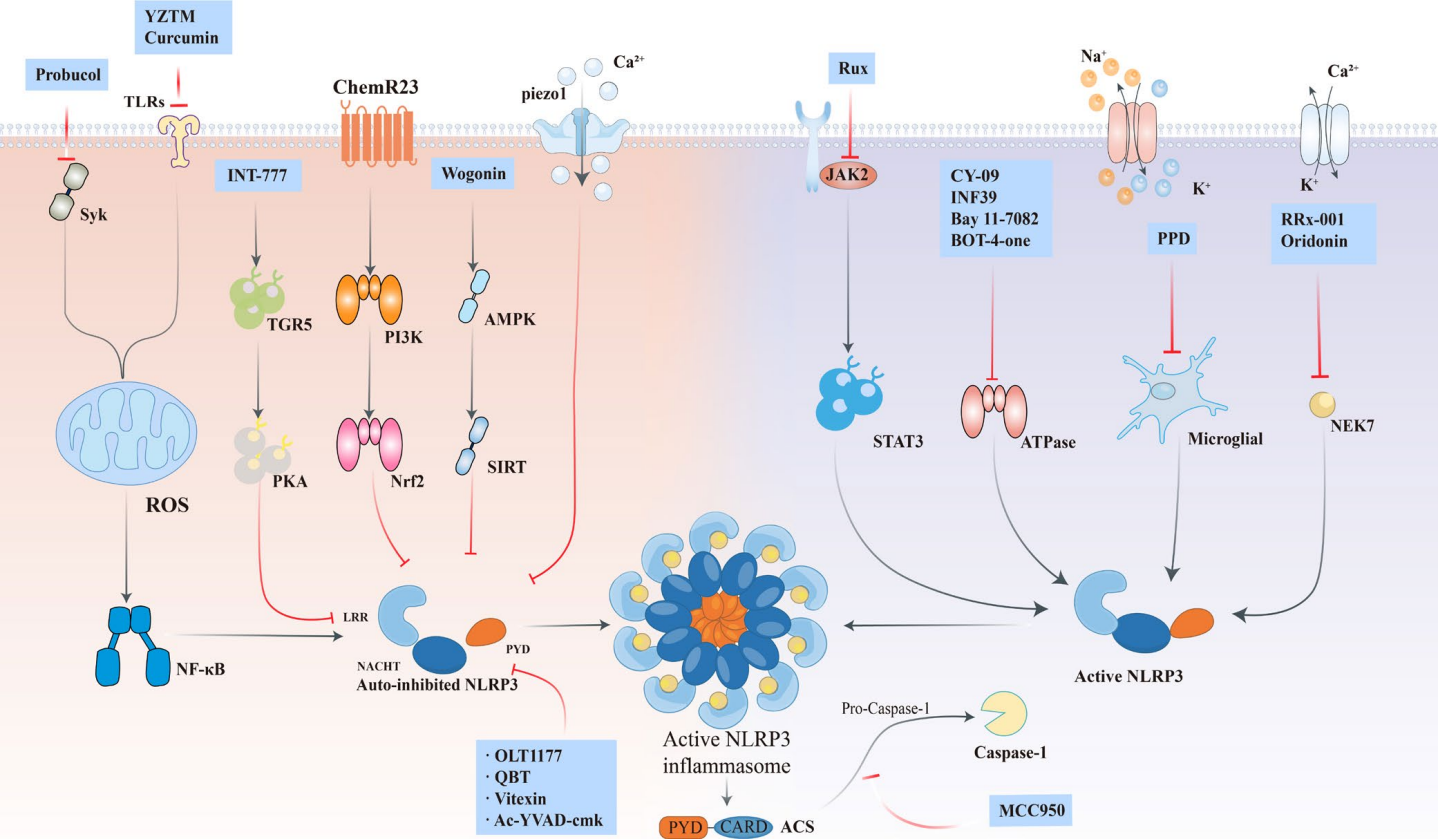
Therapeutic modalities and drugs targeting the NLRP3 inflammasome pathway. Many drugs, such as CY‐09, Bay 11‐7082, BOT‐4‐one, and INF39, block the activation of the NLRP3 ATPase activity. Other medications, such as MCC950, block the downstream binding of caspase‐1 to the PYD structural domain.

The majority of these protein targets and drugs act by preventing the activation pathway of the NLRP3 inflammasome. Therapeutic strategies targeting NLRP3 inflammasome activation encompass diverse approaches, including ROS pathway inhibition, like probucol [134], fisetin [135], and electroacupuncture [136]. Modulation of upstream signaling cascades such as JAK2/STAT3 suppression via ruxolitinib [110] and TGR5/cAMP/PKA axis inhibition by INT‐777 [137] (Figure 3). Disruption of NLRP3‐NEK7 interactions through pharmacological agents like INF39 [138], oridonin (Ori) [139], and RRx‐001 [140], direct NLRP3 targeting via tranilast (TL) [141] and acupuncture [142]. As well as inhibition of NLRP3 ATPase activity by CY‐09 [143], Bay 11‐7082 [144], BOT‐4‐one [145, 146], INF39 [138, 147], oridonin (Ori) [139] (For detailed drug profiles, refer to Table 1).

**TABLE 1:** Drugs/targets/methods targeting the NLRP3 inflammasome.

Drugs/targets/methods	Mechanism	Function	Animal models	Years	Reference
OLT1177 (dapansutrile)	NLRP3	Inhibits	ICH surgery mice	2024	[148]
QBT	NLRP3	Inhibits	VaD rats	2024	[149]
Probucol	Syk/ROS	Inhibits	2‐VO SD rats	2024	[134]
Ac‐YVAD‐cmk	NLRP3/caspase‐1/interleukin‐1β	Inhibits	BCAS mice	2024	[150]
Wogonin	AMPK/SIRT/NLRP3	Activates	MCAO rats	2024	[151]
TongqiaoYizhiKeli (TQYZKL)	Kappa‐B/NLRP3	Inhibits	2‐VO rats	2024	[152]
Piezo1 channel	Piezo1/NLRP3	Inhibits	BCCAO rats	2024	[153]
Treadmill Exercise	CX3CL1/NF‐κB/NLRP3	Inhibits	MCAO mice	2024	[113, 114]
MCC950	ASC oligomerization	Inhibits	Human stem cell population	2023	[154, 155]
METTL14	KAT3B‐STING to NLRP3/pyroptosis axis	Inhibits	MCAO rats	2023	[111]
Protopanaxadiol (PPD)	Microglial/NLRP3	Inhibits	2‐VO rats	2023	[156]
ChemR23	PI3K/AKT/Nrf2/ NLRP3	Inhibits NLRP3	CCH rat	2023	[157]
Zishen Huoxue recipe	NLRP3	Inhibits	BCCAO rats	2023	[158]
Cornel iridoid glycoside (CIG)	NLRP3/calpain signaling	Inhibits	2‐VO rats	2022	[159]
Fisetin	ROS/NF‐κB/NLRP3 AND Nrf2/HO‐1	Inhibits	Mouse model of VaD	2022	[135]
Electroacupuncture	ROS/NLRP3	Inhibits	BCCAO rats	2022	[136, 160, 161]
YZTM	NLRP3 AND TNF signaling pathway AND toll‐like receptor	Inhibits	No	2021	[162]
Ruxolitinib (Rux)	JAK2/STAT3/ NLRP3	Inhibits	MCAO mouse	2021	[110]
Ubiquitin‐specific protease 19 (USP19)	ROS/NLRP3	Activates	USP19 mice	2021	[163]
Vitexin	NLRP3	Inhibits	2‐VO rats	2021	[164]
RRx‐001	NLRP3‐NEK7	Inhibits	Nlrp3−/− mice	2021	[140]
TRPV1 channel	TRPV1‐Ca2 + ‐PP2A pathway	Activates	TRPV1‐KO (TRPV1−/−) mice	2021	[165]
Curcumin	TREM/TLR4/NF‐κB/NLRP3	Inhibits	High‐fat diet rats	2021	[166, 167]
INF39	NLRP3 ATPase activity AND NEK7‐NLRP3	Inhibits	Human THP‐1 cells	2021	[138, 147]
INT‐777	TGR5/cAMP/PKA/NLRP3	Inhibits	SAH rats	2021	[137]
Fastigial nucleus (FSN)	NLRP3	Inhibits	CCH rat	2019	[168]
Oridonin (Ori)	NEK7‐NLRP3	Inhibits	Mouse CAPS	2018	[139]
Tranilast (TR)	the NACHT domain of NLRP3 AND NLRP3 oligomerization	Inhibits	Nlrp3 −/− mice, Nlrp3 A350VneoR mice	2018	[141]
Acupuncture	ROS/TXNIP/NLRP3	Inhibits	2VO + Acu rats	2018	[142]
CY‐09	NLRP3 ATPase activity	Inhibits	CAPS and type 2 diabetes mouse	2017	[143]
BOT‐4‐one	NLRP3 ATPase activity	Inhibits	Mouse	2017	[145, 146]
DY‐9836	NLRP3	Inhibits	BCAS mice	2017	[169]

Abbreviations: 2‐VO, bilateral carotid artery occlusion; BCAS, bilateral common carotid artery stenosis; BCCAO, bilateral common carotid artery occlusion; BMDMS, bone marrow‐derived macrophages; CAPS, cryopyrin‐associated autoinflammatory syndrome; SD, Male Sprague–Dawley rats.

These medications effectively block the activation of the NLRP3 inflammasome in different models, thereby reducing neuroinflammation triggered and exacerbated by the NLRP3 inflammasome pathway. Interestingly, smoking also promotes the NLRP3 inflammasome pathway in VaD [170]. Additionally, in mouse models, the NLRP3 inflammasome inhibitor OLT1177 alleviates symptoms after ICH [148] and might also have a therapeutic effect on VaD caused by ischemic stroke. Recent research showed that INF39 inhibited the activation of the NLRP3 inflammasome in the human THP‐1 cell model [138, 147]. These preclinical studies suggest that drug therapy targeting the regulation of the NLRP3 inflammasome can improve neuroinflammation and, consequently, VaD.

### 5.2 Pathway Regulation via Secretory Factors and Cell Interactions

Neuroinflammation caused by the NLRP3 inflammasome pathway can be inhibited by blocking the transmission of factors within the pathway, as well as influencing the activation and initiation of the NLRP3 inflammasome. Currently, several substances have been discovered that can affect the protease activity of the secreted caspase‐1 and the processing of IL‐1β within the pathway. MCC950, for instance, functions during the activation phase of the NLRP3 inflammasome and blocks the processing of IL‐1β [100, 101]. Another substance is sesquiterpene lactone parthenolide, which directly inhibits the protease activity of caspase‐1 [144]. Recently, tongqiaoYizhiKeli (TQYZKL) was found to inhibit pyroptosis of hippocampal neurons in VaD rats by regulating the nuclear factor kappa‐B/NLRP3/caspase‐1 signaling pathway, thereby exerting a therapeutic effect on rat VaD [152]. Moreover, the calmodulin inhibitor DY‐9836 and AMS‐17 [171] in the BCAS mouse model can function by inhibiting signaling within the NLRP3 inflammasome. A caspase‐1 inhibitor, Ac‐YVAD‐cmk, can also inhibit signaling in the cortical part of the NLRP3 inflammasome [150].

Moreover, cell–cell interactions can regulate the NLRP3 inflammasome activity. Many drugs can reduce the activation of microglia and thereby the production of the NLRP3 inflammasomes [98]. For example, osthole (OST) was discovered in a rat model of BCCAO [172]. Quercetin (Qu) can also prevent the interaction between the NLRP3 inflammasome and mitochondrial autophagy by blocking microglial activation to alleviate neurotoxicity [173]. Additionally, autophagy inducers targeting microglial autophagy can degrade the NLRP3 inflammasome or its components. Studies have shown that combining a specific inhibitor of the NLRP3 inflammasome with an autophagy inducer targeting autophagy degradation of microglia is more effective than single therapy, as revealed in cellular and animal models of neurodegenerative diseases [174]. Furthermore, PGC‐1α, a major coregulator of gene expression in mitochondrial biogenesis, promotes autophagy and mitochondrial autophagy and reduces NLRP3 activation through ULK1 in ischemic stroke [102]. Actively regulating the overexpression of this gene might be a promising approach for future treatment by preventing the primary disease.

Interestingly, neutrophils can also be regulated. A bacterial‐derived outer membrane vesicle can inhibit the activation and iron transport of the NLRP3 inflammasome and encapsulate pioglitazone (PGZ) to facilitate its uptake by neutrophils, assisting in the treatment of ischemic stroke, a precursor of VaD, and playing a neuroprotective role [175]. Additionally, the oligodendrocyte transcription factors Pou2f1 and Nrf1 have been identified for the first time to be involved in this process, promoting nerve repair through single‐nucleus RNA sequencing (snRNA‐seq).

### 5.3 New Therapeutic Techniques

Recent research has discovered many drugs being tested on animals, such as QBT [149], probucol [150], OLT1177 [148], Ac‐YVAD‐cmk [150], and wogonin [151] (Table 1). Moreover, innovative therapies, such as nanomaterials, hydrogen, and gene editing technology, are being explored.

#### 5.3.1 Nanomaterials

Injecting nanomicelles into the common carotid artery in the MCAO rat model can significantly reduce the level of the NLRP3 inflammasome markers [126]. DY‐9836, an encapsulated nanodrug delivery system [169], inhibits NLRP3 inflammasome activation, potentially complementing nanobacteria‐assisted gene therapy. Moreover, nanoparticles can serve as carriers, playing a role in the signaling of the NLRP3 inflammasome [176, 177].

#### 5.3.2 Hydrogen

The application of magnesium silicide nanosheets in the VaD rat model has been found to release hydrogen in vivo. Hydrogen treatment significantly improves the edema of neuronal cells and can improve neurological and cognitive functions by inhibiting ROS/NLRP3/IL‐1β‐associated oxidative stress and inflammation [127], representing a novel therapeutic target.

#### 5.3.3 Magnetic Vagus Nerve Stimulation

In a rat model with myocardial injury, magnetic vagus nerve stimulation (mVNS) inhibits NLRP3‐mediated pyroptosis through the M2AChR/OGDHL/ROS axis [178]. This might also inhibit the pathway by which ROS activates the NLRP3 inflammasome in VaD.

#### 5.3.4 Gene Editing Technology

Furthermore, gene editing technology can target the NLRP3 inflammasome to treat inflammatory diseases [179]. It is also a promising approach with favorable outcomes in numerous fields [180, 181, 182, 183]. Recent studies have identified coptisine chloride as a novel METTL3 inhibitor that can alleviate the activation of the NLRP3 inflammasome by increasing the ubiquitination of NEK7 [184].

#### 5.3.5 Peptide Inhibitors

Peptide inhibitors are currently the prevailing drugs for inhibiting the activation of the NLRP3 inflammasome. They possess higher selectivity, greater potency, lower toxicity, and fewer off‐target effects [185]. NLRP3 SIRNA‐PCLS, which targets VCAM‐1‐binding peptide as a siRNA carrier in partially carotid artery ligation rats and ApoE mice, has been recently found to block LDL transcytosis by targeting the NLRP3 inflammasome [186]. This suggests a potential new therapy for atherosclerosis with a favorable effect on VaD. Studies on neuroinflammation in neurodegenerative diseases, such as AD and PH, found that dopamine can downregulate the activation of the NLRP3 inflammasome, alleviating neuroinflammation [187, 188]. Moreover, a clinical study found that exercise can inhibit neuroinflammation [189]. Dopamine might have the same effect in VaD.

### 5.4 Clinical Research Related to the NLRP3 Inflammasome

Currently, there are limited clinical trials on drugs targeting the NLRP3 inflammasome, particularly for dementia. Our discussion centers on clinical research related to NLRP3 to lay the groundwork for further studies on NLRP3 inflammasomes in VaD. This represents a promising direction for future experimental research. Clinical research indicated that oral low‐molecular‐weight NLRP3 inhibitors, tranilast (TL) [190] and RRx‐001 [191], tested in animal models, are effective and safe. A clinical study on dapansutrile (OLT1177) in patients with heart failure also showed its safety and good tolerability [192]. Inhibitors such as DFV890 [193], colchicine [194, 195, 196], ticagrelor [197], and selnoflast [198] have been experimentally shown to be well tolerated without increased risk of AEs. Similarly, no serious AE was observed with the inhibitor ZYIL1 [199]. These findings underscore the reliability of targeting the NLRP3 inflammasome for treatment (Table 1).

Furthermore, many traditional Chinese medicines, which have undergone corresponding clinical research, inhibit the NLRP3 inflammasome. A clinical study on the gelanxinning capsule (GXSC), a traditional Chinese medicine for treating inflammation including NLRP3, proved its effectiveness and safety [200]. Qingre Lishi Decodion has a significant therapeutic effect involving NLRP3 [201]. Notably, clinical studies have found that the inhibitor GDC‐2394 can cause liver injury [202]. However, this does not negate the therapeutic value of NLRP3. Therefore, clinical trials are necessary for any drug before its use. Furthermore, research on combining NLRP3 inhibitors with other drugs has shown that this combination therapy is safe and well tolerated. This conservative approach with minimal side effects represents a new direction for later stage clinical trials [191]. Interestingly, diet and exercise play a crucial role in reducing NLRP3‐related neuroinflammation. MedDiet [203] and exercise training [204] can decrease NLRP3 inflammasomes in older individuals, highlighting their significance in the treatment of VaD.

Despite the availability of numerous NLRP3 inflammasome inhibitors, significant challenges persist in identifying clinically viable candidates. While certain inhibitors demonstrate strong target specificity and therapeutic potential, notably MCC950 and CY‐09, safety concerns remain a critical barrier. MCC950 in particular shows dose‐dependent hepatotoxicity [205], suggesting future clinical applications could explore combination regimens with other agents, dose optimization strategies, or nanomaterial‐based delivery systems to circumvent hepatic exposure. Conversely, naturally derived inhibitors like curcumin and fisetin exhibit favorable safety profiles but limited therapeutic efficacy. Strategic approaches such as pharmaceutical optimization through solvent modification or combination therapy to enhance synergistic effects warrant further investigation.

### 5.5 Clinical Research Related to the NLRP3 Inflammasome

It is noteworthy that, in clinical practice, we should not only utilize the NLRP3 inflammasome as a therapeutic target pathway for drug development but also explore its potential alternative applications, such as serving as a biomarker for early diagnosis or prognostic assessment of vascular dementia (VaD). Studies have revealed that the levels of the NLRP3 inflammasome are positively correlated with disease severity in VaD patients, and its dynamic changes demonstrate high sensitivity [126, 206], indicating its feasibility as a biomarker. However, in subsequent practical research, attention should be given to distinguishing VaD from other neurodegenerative diseases, verifying the reproducibility of findings across diverse patient populations, and conducting dynamic tracking. This approach not only provides novel insights for biomarker development but also promotes the translation of NLRP3‐related research from laboratory settings to clinical applications.

In conclusion, the current therapeutic approaches and drugs that inhibit the NLRP3 inflammasome pathway primarily suppress the activation and initiation of the NLRP3 inflammasome. This suppression reduces the release of interleukin family, caspase‐1, and GSDMD molecules downstream of the NLRP3 inflammasome, thereby inhibiting their signaling, leading to cell apoptosis and alleviating brain cell necrosis. This, in turn, eases cognitive impairment. Numerous therapeutic drugs target the NLRP3 inflammasome; however, clinical trials for new drugs are limited. Specifically, there is a lack of drugs tailored for patients with VaD, necessitating further development. Drugs used to treat the NLRP3 inflammasome pathway in other neurodegenerative and vascular diseases should be explored for their efficacy in VaD. We should leverage clinical research on other diseases and conduct targeted clinical trials for vascular dementia. Notably, dapansutrile (OLT1177), which has undergone clinical research for myocardial infarctions, is now being tested for ICH and further developed for vascular dementia.

## 6 Conclusion and Prospect

The NLRP3 inflammasome is a crucial factor in the onset and development of VaD. Currently, the NLRP3 inflammasome has been found to function by causing and exacerbating neuroinflammation, leading to the apoptosis of nerve cells. Despite the various activation pathways of the NLRP3 inflammasome, its mechanism in VaD requires further exploration. New therapeutic techniques, such as nanomaterials, hydrogen [127], magnetic vagus nerve stimulation, and gene editing, have been discovered. Additionally, blocking targets of the NLRP3 inflammasome like the ChemR23 receptor and TRPV1 channel, as well as interventions such as cerebellar electrical incision nuclear stimulation (FNS), acupuncture, and drugs like curcumin, dapansutrile (OLT1177), tranilast (TL), and parthenolide, are under investigation.

Despite the extensive research on medications, the specific mechanisms of VaD remain incompletely understood. Bioinformatics analysis can help identify additional factors with differential effects, facilitating the exploration of more detailed and specific mechanisms unique to VaD. Furthermore, we should investigate whether the NLRP3 inflammasome in VaD has alternative pathways beyond causing cell apoptosis, such as influencing anti‐inflammatory responses [82]. Currently, many treatments are based on traditional Chinese medicine and material research, with some success. However, most drug treatments for VaD remain primarily at the animal model level, with limited clinical trials. Future research should focus on advancing clinical studies in this area.

In brief, research on the NLRP3 inflammasome in VaD is highly promising and necessary. We should identify more NLRP3 inflammasome blockers that can treat VaD, develop new research methods, compare their therapeutic effects, and further explore the NLRP3 inflammasome to unveil its complexities.

## Author Contributions

The initial idea for this review was conceived by X.Z., Y.L., and L.C., and the manuscript was written and revised by Y.L. and L.C. In addition, Y.X., C.H., and Z.L. contributed to the preparation of the illustrations; C.S., H.W., and Y.Q. edited the manuscript; Y.L., S.Y., M.W., and X.Z. read, reviewed, and approved the final manuscript. The final version of the manuscript was approved by all the authors.

## Ethics Statement

The authors have nothing to report.

## Consent

The authors have nothing to report.

## Conflicts of Interest

The authors declare no conflicts of interest.

## Data Availability

Data sharing not applicable to this article as no datasets were generated or analysed during the current study.
